# Supplementary material for: Modulation of NK Cell Properties by ESKAPE Group Bacteria
Source: Int J Mol Sci. 2025 Aug 30;26(17):8449. doi: 10.3390/ijms26178449 (PMC12428669; doi:10.3390/ijms26178449)
Supplement: Supplementary file 1 [file ijms-26-08449-s001.zip › ijms-3831150-supplementary.pdf]

Table S1. Amount of amplification cycles required for PCR product detection.

	TLR1	TLR2	TLR4	TLR5	TLR6	GapdH
NK-92	28,1	29,8	nd	27,8	31,1	27,5
NK-92	27,5	26,3	nd	27,6	31,1	27,6
NK-92	27,4	26,7	nd	28,7	30,4	26,6
NK-92	27,4	26,7	nd	27,8	30	27,6
NK-92	27,7	25	nd	29	30	17,9
NK-92	27,3	25,1	nd	29	30,4	18,2
NK-92	28	27	nd	28,4	29,7	18,1
NK-92	27,7	27,2	nd	28,7	29,6	18
